# The role of geriatric syndromes in predicting unplanned hospitalizations: a population-based study using Minimum Data Set for Home Care

**DOI:** 10.1186/s12877-023-04408-w

**Published:** 2023-10-26

**Authors:** Jukka Rönneikkö, Heini Huhtala, Harriet Finne-Soveri, Jaakko Valvanne, Esa Jämsen

**Affiliations:** 1 Faculty of Medicine and Health Technology, Tampere University, Tampere, Finland; 2 Faculty of Social Sciences, Tampere University, Tampere, Finland; 3 Finnish Institute for Health and Welfare, Helsinki, Finland; 4 Faculty of Medicine and Health Technology and Gerontology Research Center (GEREC), Tampere University, Tampere, Finland; 5 Faculty of Medicine, University of Helsinki, Helsinki, Finland; 6 Department of Geriatrics, Helsinki University Hospital, Helsinki, Finland

**Keywords:** MDS-HC, Home care, Assessment, Hospitalization, Case finding tool

## Abstract

**Background:**

The predictive accuracies of screening instruments for identifying home-dwelling old people at risk of hospitalization have ranged from poor to moderate, particularly among the oldest persons. This study aimed to identify variables that could improve the accuracy of a Minimum Data Set for Home Care (MDS-HC) based algorithm, the Detection of Indicators and Vulnerabilities for Emergency Room Trips (DIVERT) Scale, in classifying home care clients’ risk for unplanned hospitalization.

**Methods:**

In this register-based retrospective study, factors associated with hospitalization among home care clients aged ≥ 80 years in the City of Tampere, Finland, were analyzed by linking MDS-HC assessments with hospital discharge records. MDS-HC determinants associated with hospitalization within 180 days after the assessment were analyzed for clients at low (DIVERT 1), moderate (DIVERT 2–3) and high (DIVERT 4–6) risk of hospitalization. Then, two new variables were selected to supplement the DIVERT algorithm. Finally, area under curve (AUC) values of the original and modified DIVERT scales were determined using the data of MDS-HC assessments of all home care clients in the City of Tampere to examine if addition of the variables related to the oldest age groups improved the accuracy of DIVERT.

**Results:**

Of home care clients aged ≥ 80 years, 1,291 (65.4%) were hospitalized at least once during the two-year study period. Unplanned hospitalization occurred following 15.9%, 22.8%, and 33.9% MDS-HC assessments with DIVERT group 1, 2–3 and 4–6, respectively. Infectious diseases were the most common diagnosis within each DIVERT groups.

Many MDS-HC variables not included in the DIVERT algorithm were associated with hospitalization, including e.g. poor self-rated health and old fracture (other than hip fracture) *(p 0.001)* in DIVERT 1; impaired cognition and decision-making, urinary incontinence, unstable walking and fear of falling (*p* < *0.001*) in DIVERT 2–3; and urinary incontinence, poor self-rated health (*p* < *0.001*), and decreased social interaction (*p 0.001*) in DIVERT 4–6*.* Adding impaired cognition and urinary incontinence to the DIVERT algorithm improved sensitivity but not accuracy (AUC 0.64 (95% CI 0.62–0.65) vs. 0.62 (0.60–0.64) of the original DIVERT). More admissions occurred among the clients with higher scores in the modified than in the original DIVERT scale.

**Conclusions:**

Certain geriatric syndromes and diagnosis groups were associated with unplanned hospitalization among home care clients at low or moderate risk level of hospitalization. However, the predictive accuracy of the DIVERT could not be improved. In a complex clinical context of home care clients, more important than existence of a set of risk factors related to an algorithm may be the various individual combinations of risk factors.

**Supplementary Information:**

The online version contains supplementary material available at 10.1186/s12877-023-04408-w.

## Background

Old age is often associated with a decline in health and functional abilities as a result of multimorbidity, frailty, and age-related physiological changes [1], and a growing number of older people requires help in daily life to survive at home [2]. This has led to an effort to develop different services to compensate for the loss of functional capacity and to enable old people with functional limitations to live in their own homes longer [3].

Home care services are one way to support older people with functional limitations, ensuring adequate assistance in everyday activities when individuals can no longer manage alone owing to physical or cognitive impairment and diseases. From clinical point of view, home care clients are in a complex clinical situation. They are old (majority aged ≥ 75 years), have several chronic conditions and associated polypharmacy (at least in half of the clients) and most clients have some level of cognitive impairment, every sixth having moderate to severe impairment [4, 5]. Incontinence, falls, and loneliness are also common, and unfortunately a large part of home care clients experience a decline in ADL while receiving home care services [4]. For these reasons, home care clients are predisposed to many kinds of different adverse outcomes, such as unplanned hospitalizations and emergency room visits [6]. Among home care clients, the rate of hospitalization has ranged from 16–38% in 2–6-month to 15–48% in one-year follow-up [7–13]. Indeed, in a Finnish study, nearly half of patients in primary health care hospital wards were home care clients [14] and were, in most cases, hospitalized due to acute reasons [15].

For old people, unplanned hospitalization may lead to hospital care-related adverse events and cause new functional deficits [16, 17]. Prediction of older persons at risk of hospital admissions provides an opportunity to target health care interventions, such as comprehensive geriatric assessment (CGA), a multidimensional, multidisciplinary diagnostic and therapeutic process for determining an older person’s medical, psychological, and functional capabilities. CGA can be used to develop a patient-centered, coordinated and integrated care plan including long-term follow-up [18–20].

To identify patients at risk of unplanned hospitalizations, several risk prediction models have been developed [21–30]. The most frequently incorporated predictors have been medical comorbidities, age, previous healthcare utilisation [29, 31], and self-rated quality of life [31]. The reported accuracies have ranged from poor to moderate accuracy, depending on the assessment tool, population, setting and follow-up. Although 25–30% of persons aged 80 years or older accessing emergency care settings are frail [32] and frailty has been associated with a high risk of multiple adverse health outcomes [32–37], the usefulness of frailty scales in classifying the risk of unplanned hospitalization is unclear [38].

The Detection of Indicators and Vulnerabilities for Emergency Room Trips (DIVERT) Scale is a valid case-finding algorithm for classifying the risk of emergency department (ED) visits in older home care clients utilizing Minimum Data Set for Home Care (MDS-HC) instrument [39–41]. In an earlier study [42], we observed relatively low predictive accuracy for the DIVERT scale in classifying the risk of unplanned hospitalization among home care clients especially in the oldest age groups. A similar result was obtained in a cross-country external validation study, where DIVERT showed substantial variations from poor to fair performance in predicting unplanned hospitalization across European countries [41].

Based on earlier studies [13, 43], there are geriatric symptoms and syndromes that predict unplanned hospitalization of old people but are not included in DIVERT or other screening tools. If they are associated with hospitalizations, DIVERT may underestimate the risk of hospitalization particularly among clients with low or moderate risk of hospitalization. This could reduce the accuracy of DIVERT scale in risk prediction, especially among the oldest home care clients.

The first aim of this study was to identify variables that are not included in the DIVERT scale but are associated with hospitalizations in the oldest home clients aged ≥ 80 years. The second aim was to test if the accuracy of the DIVERT scale among home care clients of all ages could be improved by adding such variables to the algorithm.

## Materials and methods

Minimum Data Set for Home Care (MDS-HC), a predecessor of interRAI-HC assessment tool (an assessment system developed by the InterRAI research network [44]), is a comprehensive assessment tool, identifying the needs of home care clients with disabilities and collecting data for a comprehensive assessment from function, health, social support, and service use [45]. Its reliability and validity have been tested in international studies [45–47]. In addition to the DIVERT Scale, the MDS-HC scales measuring activities of daily living performance (ADLh) [48], cognitive performance (CPS) [49], depression (DRS) [50], pain (PAIN) [51], and health stability (CHESS) [52] were used in this study. All used MDS-HC variables are listed in Table 1.

**Table 1:** Characteristics of those who were hospitalized in each DIVERT group

**Client characteristics**		**DIVERT**		**DIVERT**		**DIVERT**		
	**ALL**	**1**		**2–3**		**4–6**		
	**N**	**N**	**%**	**N**	**%**	**N**	**%**	***p***
	**1291**	**137**	10.6	**536**	41.5	**618**	47.9	** < *.001***
**Demographic**								
Age								*.965*
80–89	**915**	**98**	10.7	**381**	41.6	**436**	47.7	
90 +	**376**	**39**	10.4	**155**	41.2	**182**	48.4	
Gender								*.364*
Male	**300**	**33**	11.0	**114**	38.0	**153**	51.0	
Female	**991**	**104**	10.5	**422**	42.6	**465**	46.9	
**Social situation**								
Caregiver stressed	**92**	**4**	4.3	**41**	44.6	**47**	51.1	*.131*
Housing-related problems	**362**	**26**	7.2	**145**	40.1	**191**	52.8	*.015*
**Use or needs of services**								
Reason for home care: client has been discharged from hospital	**518**	**42**	8.1	**195**	37.6	**281**	54.2	< *.001*
Acute outpatient care or unplanned hospitalization in 90 days before assessment^a^	**629**	**0**	0.0	**80**	12.7	**549**	87.3	< *.001*
**Function**								
Activities of Daily Living Hierarchy score (0—6)								*.056*
0	**1137**	**129**	11.3	**472**	41.5	**536**	47.1	
1–2	**91**	**5**	5.5	**44**	48.4	**42**	46.2	
3–4	**36**	**1**	2.8	**10**	27.8	**25**	69.4	
5–6	**26**	**2**	7.7	**9**	34.6	**15**	57.7	
ADL decline in previous 90 days^a^	**578**	**0**	0.0	**258**	44.6	**320**	55.4	< *.001*
Poor prospects for functional improvement^a^	**99**	**7**	7.1	**42**	42.4	**50**	50.5	*.486*
Client doesn't believe he/she is capable of improving performance in physical function	**861**	**92**	10.7	**373**	43.3	**396**	46.0	*.139*
Cognitive Performance Scale score (0–6)								< *.001*
0	**296**	**39**	13.2	**95**	32.1	**162**	54.7	
1–2	**798**	**91**	11.4	**334**	41.9	**373**	46.7	
3–4	**134**	**5**	3.7	**70**	52.2	**59**	44.0	
5–6	**63**	**2**	3.2	**37**	58.7	**24**	38.1	
Impairment of decision-making capacity	**423**	**13**	3.1	**194**	45.9	**216**	51.1	< *.001*
Decreased social interaction	**244**	**6**	2.5	**88**	36.1	**150**	61.5	< *.001*
Worsening of social interaction	**500**	**31**	6.2	**224**	44.8	**245**	49.0	< *.001*
**Clinical symptoms**								
Any cardiorespiratory symptoms^a^	**824**	**0**	0.0	**316**	38.3	**508**	61.7	< *.001*
Urinary incontinence daily	**438**	**33**	7.5	**191**	43.6	**214**	48.9	*.034*
Worsening of urinary incontinence	**304**	**12**	3.9	**142**	46.7	**150**	49.3	< *.001*
Urinary catheter^a^	**17**	**2**	11.8	**5**	29.4	**10**	58.8	*.588*
Faecal incontinency weekly	**99**	**10**	10.1	**44**	44.4	**45**	45.5	*.831*
Chronic skin ulcers	**112**	**5**	4.5	**47**	42.0	**60**	53.6	*.074*
Stasis ulcer^a^	**167**	**0**	0	**85**	50.9	**82**	49.1	< *.001*
Mouth problems	**322**	**21**	6.5	**132**	41.0	**169**	52.5	*.013*
Vision								*.727*
Good enough	**838**	**94**	11.2	**345**	41.2	**399**	47.6	
Moderately impaired	**399**	**36**	9.0	**171**	42.9	**192**	48.1	
Severely impaired	**54**	**7**	13.0	**20**	37.0	**27**	50.0	
Falls during 90 days before assessment^a^	**388**	**0**	0.0	**163**	42.0	**225**	58.0	< *.001*
Unstable walking	**1062**	**100**	9.4	**446**	42.0	**516**	48.6	*.011*
Fear of walking	**788**	**70**	8.9	**339**	43.0	**379**	48.1	*.033*
Depression Rating Scale score 0–14)								< *.001*
0–2	**1047**	**127**	12.1	**455**	43.5	**465**	44.4	
3–14	**244**	**10**	4.1	**81**	33.2	**153**	62.7	
Any mood symptoms^a^	**656**	**33**	5.0	**247**	37.7	**376**	57.3	< *.001*
Increased mood symptoms in 90 days	**283**	**10**	3.5	**100**	35.3	**173**	61.1	< *.001*
Any behavioural symptom	**198**	**11**	5.6	**82**	41.4	**105**	53.0	*.031*
Worsening of behavioural symptoms in 90 days	**109**	**6**	5.5	**44**	40.4	**59**	54.1	*.140*
Alcohol abuse	**19**	**1**	5.3	**7**	36.8	**11**	57.9	*.599*
Feeling lonely	**375**	**27**	7.2	**159**	42.4	**189**	50.4	*.037*
Poor self-rated health	**461**	**33**	7.2	**162**	35.1	**266**	57.7	< *.001*
Weight loss^a^	**66**	**0**	0.0	**19**	28.8	**47**	71.2	< *.001*
Body mass index, kg/m2								*.224*
< 18.5	**55**	**7**	12.7	**24**	43.6	**24**	43.6	
18.5–23.9	**405**	**47**	11.6	**149**	36.8	**209**	51.6	
24–29.9	**538**	**56**	10.4	**226**	42.0	**256**	47.6	
30	**248**	**20**	8.1	**117**	47.2	**111**	44.8	
Decrease in food or fluids^a^	**64**	**0**	0.0	**31**	48.4	**33**	51.6	*.017*
Pain Scale (0–3)								< *.001*
0–1	**698**	**95**	13.6	**312**	44.7	**291**	41.7	
2–3	**593**	**42**	7.1	**224**	37.8	**327**	55.1	
**Diagnoses**^**b**^								
Congestive heart failure^a^	**413**	**18**	4.4	**141**	34.1	**254**	61.5	< *.001*
Coronary artery disease^a^	**428**	**38**	8.9	**139**	32.5	**251**	58.6	< *.001*
Alzheimer's disease	**417**	**41**	9.8	**200**	48.0	**176**	42.2	*.005*
Other dementia	**156**	**18**	11.5	**67**	42.9	**71**	45.5	*.804*
History of stroke^a^	**69**	**10**	14.5	**27**	39.1	**32**	46.4	*.557*
Parkinson's disease	**32**	**4**	12.5	**12**	37.5	**16**	50.0	*.874*
Musculoskeletal disorders	**167**	**14**	8.4	**69**	41.3	**84**	50.3	*.567*
Old hip fracture	**53**	**4**	7.5	**18**	34.0	**31**	58.5	*.279*
Old other fracture	**60**	**8**	13.3	**21**	35.0	**31**	51.7	*.529*
Psychiatric diagnosis	**203**	**21**	10.3	**85**	41.9	**97**	47.8	*.988*
Chronic obstructive pulmonary disease^a^	**159**	**5**	3.1	**44**	27.7	**110**	69.2	< *.001*
Cancer	**120**	**12**	10.0	**47**	39.2	**61**	50.8	*.792*
Diabetes^a^	**398**	**39**	9.8	**159**	39.9	**200**	50.3	*.501*
Pneumonia^a^	**48**	**2**	4.2	**14**	29.2	**32**	66.7	< *.001*
History of urinary tract infection	**98**	**11**	11.2	**20**	20.4	**67**	68.4	< *.001*
Renal insufficiency^a^	**173**	**10**	5.8	**41**	23.7	**122**	70.5	< *.001*
**Medication**								
Number of drugs^c^								< *.001*
0–4	**74**	**12**	16.2	**37**	50.0	**25**	33.8	
5–8	**339**	**53**	15.6	**166**	49.0	**120**	35.4	
9 or more	**878**	**72**	8.2	**333**	37.9	**473**	53.9	
Psychotropic medication	**749**	**63**	8.4	**303**	40.5	**383**	51.1	*.002*
Influenza vaccination	**647**	**64**	9.9	**266**	41.1	**317**	49.0	*.598*
**Special therapies**								
Oxygen therapy^a^	**22**	**0**	0.0	**0**	0.0	**22**	100.0	< *.001*
**Health stability**								
Changes in Health, End-Stage Disease, Signs, and Symptoms Scale score (0–5)								< *.001*
0	**367**	**103**	28.1	**142**	38.7	**122**	33.2	
1	**378**	**33**	8.7	**172**	45.5	**173**	45.8	
2–5	**546**	**1**	0.2	**222**	40.7	**323**	59.2	

^a^Variables included in the DIVERT algorithm

^b^All previously diagnosed diseases although they do not require monitoring or treatment at the time of assessment, and diagnoses as a reason for hospitalization in 90 days prior to assessment

^c^Including prescription and non-prescription medications

The six-level DIVERT Scale, originally developed to classify the risk of ED admission in older home care clients, is an algorithm generated from MDS-HC data and includes previous ED use, cardiorespiratory symptoms, cardiac conditions, diagnoses of stroke, diabetes, renal failure, pneumonia, chronic obstructive pulmonary disease, and urinary tract infection and certain geriatric symptoms and syndromes: mood symptoms, falls, poor nutrition, skin ulcers, and ADL decline [39]. Like the standard MDS-HC scales, higher scores indicate a worse condition. Due to the limited case numbers for certain variables (for example functional and cognitive impairment, poor prospects for functional improvement, weight loss, and some specific diagnoses) there was a need to combine DIVERT levels into three categories describing the risk of hospitalization: DIVERT 1 low, 2–3 moderate, and 4–6 high risk of hospitalization to ensure a sufficient number of events at each risk level studied.

**Fig. 1:**
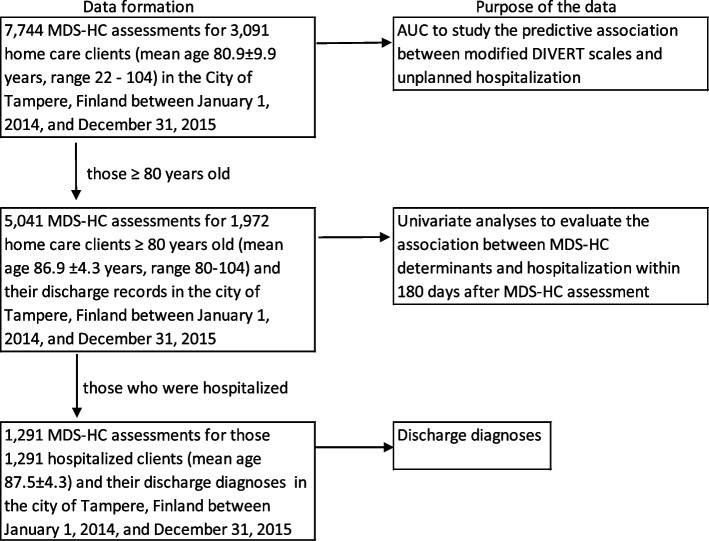
The data formation

The study was based on the data of the MDS-HC index assessments (n = 5,041) of 1,972 home care clients aged ≥ 80 years (mean age 86.9 ± 4.3 years, range 80–104) and the discharge records of those hospitalized, in the city of Tampere, Finland (ca. 240,000 inhabitants, of which 5% are aged ≥ 80 years) between January 1, 2014, and December 31, 2015. The data is a part of the original data of 7,744 RAI-HC assessments for 3,091 home care clients (mean age 80.9 ± 9.9 years, range 22–104) used in our earlier study [42]. The data formation is presented in Fig. 1.

In Finland, communities are responsible for arranging home care services to their citizens who are not able to manage activities of daily living independently, according to their needs regardless of the time of day. Home care encompasses home services, home nursing, and support services such as meals, hygiene, transport, cleaning, escort, commercial and security services, and the promotion of social interaction [53]. The client's condition is monitored at all home care visits, and a more extensive assessment of the client should be carried out every six months. Home care nurses are supported by dedicated home care physicians or general practitioners in the area. The MDS-HC assessments are carried out by a trained nurses who know the client's condition best and the assessments are a part of their normal work. The manual for MDS-HC assessments provides guidelines and definitions for completing the RAI-HC assessments with the same criteria for all clients.

The outcome was an unplanned hospitalization within 180 days after each MDS-HC assessment (according to national guidelines, home care clients are assessed upon initiation of services and thereafter at least twice a year or when there is a significant change in the client’s health or social condition). If a client had multiple MDS-HC assessments available, they were all included and considered as separate events and hospitalizations within 180 days after each assessment were recorded. However, if a client met the outcome (i.e. experienced an unplanned hospitalization), his/her all later MDS-HC assessments were ignored.. The follow-up time was the same as in our previous study concerning the accuracy of the DIVERT scale in classifying the risk of home care client for unplanned hospitalization [42], allowing comparison of the research results. As in our previous study, the hospitalizations were identified from the mandatory hospital discharge records of Tampere University Hospital and the secondary and primary care wards of the City of Tampere representing public health care and covering all unplanned inpatient care within the area regardless of social or insurance status, and were linked to the MDS-HC data using each patient’s unique identification number [42]. Scheduled hospitalizations (e.g. elective surgery) were not considered. If a client met the outcome, he/she was excluded from further follow-up and later MDS-HC assessments were ignored. If later hospital admissions had also been taken into account, it would have been difficult to make differences between the effects of patient characteristics and the consequences of the previous hospitalizations on the reasons of a new hospital episode.

As in our previous study, the discharge diagnoses were divided into nine diagnosis groups according to the first registered diagnosis (representing the main cause of hospitalization according to the treating physician): infectious diseases; dementia; cardiovascular, cerebrovascular, and musculoskeletal diseases; other specific diseases; geriatric symptoms (e.g. malaise, dizziness, syncope, malnutrition); injuries; and other reasons [43] (using International Classification of Diagnoses, 10^th^ revision) (Additional file 1).

Of the geriatric symptoms, syndromes or diagnoses, that have been shown to be independent risk factors for unplanned hospitalization in our previous studies but that are not included in DIVERT algorithm (polypharmacy, daily urinary incontinence, faecal incontinence, cognitive impairment, housing-related problems, poor self-rated health, Parkinson’s disease, and cancer [13, 43]), and were associated (*p* ≤ 0.001 in univariate analysis) with the risk of hospitalization among clients at moderate or low risk levels of DIVERT in the present study, two clinically relevant variables were selected to supplement the original DIVERT algorithm.

**Fig. 2:**
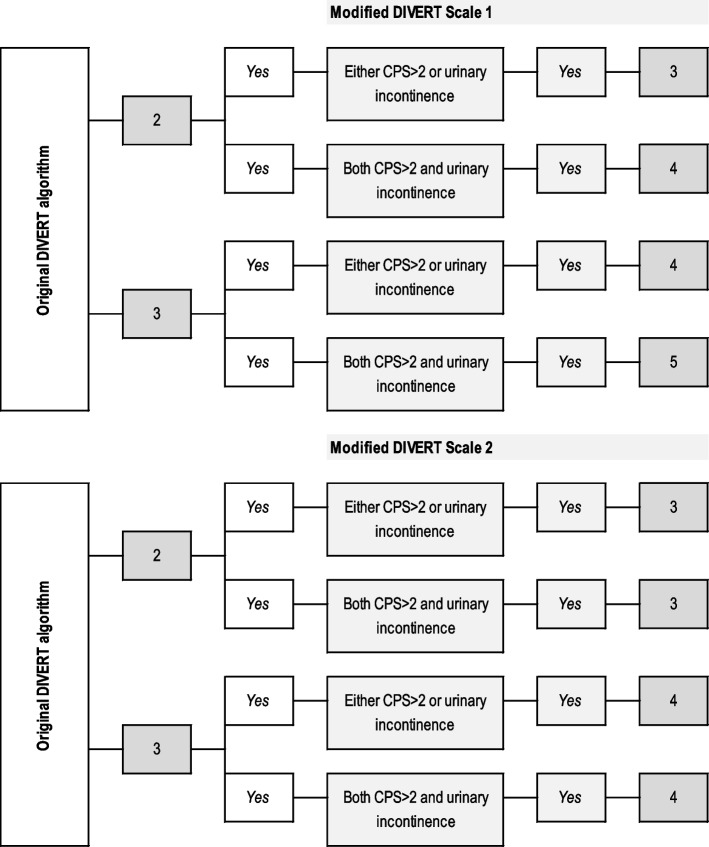
Modification of the DIVERT scale

In modifying the DIVERT algorithm, two different ways were tested. First, the risk level of DIVERT scale was increased from the original by one step, if either of the selected variables was present, and by two steps if both were present. Secondly, the increase was only one step when either one or both variables were present (Fig. 2). In both options, the aim was to redirect those with these risk factors of later hospitalization to a higher risk level in the DIVERT algorithm.

When the predictive association between the modified DIVERT scales and unplanned hospitalization were studied, the original data of 3,091 home care clients and their MDS-HC assessments (*n* = 7,744) were used. The aim was to test if the accuracy of the DIVERT scale improved, when the characteristics of the oldest clients were taken into account in the algorithm.

### Statistical analyses

Univariate binary logistic regression analysis was used to explore which MDS-HC variables were associated with hospitalization in each DIVERT group. The results are presented as odds ratios (OR) with 95% confidence intervals (CI). Significances were defined as *p*-values and *p* ≤ 0.05 was considered statistically significant.

To determine the accuracy of the modified DIVERT scales, Receiver Operating Character Curves (ROC) were calculated using the whole data including all 3,091 home care clients and their 7,744 MDS-HC assessments. The areas under the receiver operating characteristic curve (AUC) and sensitivity and specificity of the modified DIVERT scales are presented for the whole data and separately for different age groups (< 70, 70–79, 80–89, ≥ 90 years) and are compared to figures observed in our previous work concerning DIVERT scale in classifying the risk for hospitalization among home care clients [42]. The statistical analyses were performed using SPSS version 25 (IBM Corp, Armonk, NY).

## Results

Of the 5,041 MDS-HC assessments (for 1,972 home care clients), 1,291 (25.6%) were followed by an unplanned hospitalization during the 180 days after the assessment, meaning that 65.4% of the clients were hospitalized at least once during the study period of two years. Of the assessments, 3,857 (76.5%) were in the age group 80–89 years and 1,184 (23.5%) in ≥ 90 years. Of the 1,291 clients hospitalized, 915 (70.9%) were 80–89 years and 376 (29.1%) ≥ 90 years and their mean age was 87.5 ± 4.3 years. Unplanned hospitalization occurred following 15.9%, 22.8%, and 33.9% MDS-HC assessments with DIVERT group 1, 2–3 and 4–6, respectively. The characteristics of those who were hospitalized in each DIVERT group are described in Table 1.

Gender and age distribution were similar in every group. Previous discharge from hospital was a common reason for the beginning of home care services in all groups, being the most common 45.5% in the DIVERT group 4–6 (vs. 30.7% and 36.4% in the DIVERT groups 1 and 2–3, respectively). Of all hospitalized clients, 137 (10.6%) were classified in the DIVERT group 1 (low risk), 536 (41.5%) in the DIVERT group 2–3 (moderate risk), and 618 (47.9%) in the DIVERT group 4–6 (high risk) based on the MDS-HC assessment prior to the hospitalization.

The three most common specific reasons for hospitalization were infectious diseases (22.9%; *n* = 295), cardiovascular diseases (14.5%; *n* = 187) and dementia diseases (12.5%; *n* = 162). Infectious diseases were also the most common diagnosis in each DIVERT group (Table 2).

**Table 2:** The distribution of diagnoses in different DIVERT groups (*p* = 0.011)

	**ALL**		**DIVERT 1**		**DIVERT 2–3**		**DIVERT 4–6**	
	**N**	**%**	**N**	**%**	**N**	**%**	**N**	**%**
Infectious diseases	**295**	22.9	**27**	19.7	**119**	22.2	**149**	24.1
Dementia diseases	**162**	12.5	**20**	14.6	**77**	14.4	**65**	10.5
Cardiovascular diseases	**187**	14.5	**21**	15.3	**64**	11.9	**102**	16.5
Cerebrovascular diseases	**26**	2.0	**3**	2.2	**9**	1.7	**14**	2.3
Musculoskeletal diseases	**73**	5.7	**5**	3.6	**30**	5.6	**38**	6.1
Injuries	**135**	10.5	**19**	13.9	**68**	12.7	**48**	7.8
Other specific diseases	**138**	10.7	**11**	8.0	**54**	10.1	**73**	11.8
Geriatric symptoms	**107**	8.3	**13**	9.5	**49**	9.1	**45**	7.3
Other diseases and symptoms	**168**	13.0	**18**	13.1	**66**	12.3	**84**	13.6

Urinary tract infections (*n* = 136) and Alzheimer’s disease (*n* = 133) were the two most common individual diagnosis accounting for both about 10% of all discharge diagnoses.

### Univariate analysis

In the univariate analysis, many of the analyzed variables not included in the DIVERT algorithm, were associated with hospitalization (Additional file 2). Some of the factors had similar effect in all DIVERT groups but there were also differences. The factors associated with hospitalization most significantly were poor self reported health and old fracture (other than hip fracture) *(p* = *0.001)* in DIVERT 1; impaired cognition and decision-making, worsening of social interaction, urinary incontinence and it’s worsening, unstable walking, fear of falling ( *p* < *0.001*) and client’s belief he/she isn’t capable of improving performance in physical function (*p* = *0.001*) in DIVERT 2–3; and urinary incontinence, poor self reported health condition, ( *p* < *0.001*) and decreased social interaction (*p* = *0.001)* in DIVERT 4–6 (Table 3).

**Table 3:** The univariate analysis showing the associations between MDS-HC -determinants and hospitalization (*p* < .05 in any DIVERT group) during the follow up of 180 days from index MDS-HC assessment at different DIVERT levels

	**DIVERT 1**			**DIVERT 2–3**			**DIVERT 4–6**		
	**Univariate**			**Univariate**			**Univariate**		
	**OR**	**95% CI**	***p***	**OR**	**95% CI**	***p***	**OR**	**95% CI**	***p***
**Demographic**									
Age									
80–89	**1**			**1**			**1**		
90 +	**1.91**	1.26–2.90	*.002*	**1.39**	1.12–1.73	*.003*	**1.44**	1.16–1.80	*.001*
**Social situation**									
Caregiver stressed^a^	**1.33**	0.43–4.08	*.623*	**1.52**	1.03–2.23	*.034*	**1.33**	0.90–1.96	*.150*
**Use or needs of services**									
**Function**									
Activities of Daily Living Hierarchy score (0—6)									
0	**1**			**1**			**1**		
1–2	**4.51**	1.36–15.0	*.014*	**2.08**	1.41–3.05	< *.001*	**1.87**	1.22–2.87	*.004*
3–4	**0.42**	0.05–3.21	*.400*	**1.20**	0.58–2.46	*.629*	**2.27**	1.28–4.04	*.005*
5–6	**1.55**	0.32–7.53	*.590*	**1.99**	0.84–4.29	*.123*	**1.84**	0.91–3.72	*.088*
ADL decline in previous 90 days^a^	Didn’t appear at level 1			**1.42**	1.17–1.73	< *.001*	**1.6**	1.32–1.95	< *.001*
Client doesn't believe he/she is capable of improving performance in physical function	**1.25**	0.85–1.84	*.255*	**1.43**	1.16–1.76	*.001*	**1.27**	1.04–1.55	*.019*
Cognitive Performance Scale score (0–6)									
0	**1**			**1**			**1**		
1–2	**1.19**	0.79–1.79	*.403*	**1.52**	1.18–1.96	*.001*	**1.08**	0.86–1.35	*.498*
3–4	**0.87**	0.32–2.35	*.780*	**2.14**	1.50–3.05	< *.001*	**1.37**	0.94–1.99	*.105*
5–6	**1.31**	0.27–6.30	*.736*	**3.24**	2.03–5.17	< *.001*	**1.46**	0.84–2.54	*.179*
Impairment of decision-making capacity	**0.96**	0.52–1.80	*.909*	**1.44**	1.18–1.77	< *.001*	**1.28**	1.04–1.57	*.019*
Decreased social interaction	**0.56**	0.24–1.32	*.184*	**1.22**	0.93–1.58	*.147*	**1.52**	1.20–1.93	*.001*
Worsening of social interaction	**1.03**	0.67–1.60	*.891*	**1.45**	1.19–1.76	< *.001*	**1.2**	0.98–1.47	*.074*
**Clinical symptoms**									
Any cardiorespiratory symptoms^a^	Didn’t appear at level 1			**0.84**	0.69–1.02	*0.081*	**1.50**	1.18–1.92	*.001*
Urinary incontinence daily	**1.12**	0.73–1.71	*.620*	**1.60**	1.20–1.97	< *.001*	**1.56**	1.26–1.92	< *.001*
Worsening of urinary incontinence	**1.01**	0.53–1.92	*.983*	**1.52**	1.21–1.90	< *.001*	**1.3**	1.03–1.64	*.028*
Faecal incontinency weekly	**2.53**	1.17–5.48	*.018*	**1.29**	0.90–1.85	*.168*	**1.17**	0.80–1.71	*.424*
Chronic skin ulcers	**2.71**	0.91–8.04	*.073*	**1.85**	1.28–2.66	*.001*	**2.06**	1.42–2.98	< *.001*
Stasis ulcer^a^	Didn’t appear at level 1			**2.01**	1.28–3.16	*.003*	**2.13**	1.36–3.32	*.001*
Falls during 90 days before assessment^a^	Didn’t appear at level 1			**1.35**	1.09–1.67	*.005*	**1.11**	0.90–1.35	*.335*
Unstable walking	**1.73**	1.16–2.60	*.008*	**1.68**	1.31–2.16	< *.001*	**1.42**	1.10–1.82	*.007*
Fear of walking	**1.35**	0.94–1.95	*.109*	**1.55**	1.27–1.89	< *.001*	**1.36**	1.12–1.66	*.002*
Depression Rating Scale score 0–14)									
0–2	**1**			**1**			**1**		
3–14	**1.22**	0.60–2.48	*.589*	**1.33**	1.01–1.75	*.044*	**1.33**	1.06–1.68	*.015*
Any mood symptoms^a^	**0.80**	0.52–1.22	*.296*	**1.36**	1.12–1.65	*.002*	**1.49**	1.22–1.82	< *.001*
Increased mood symptoms in 90 days	**1.42**	0.69–2.93	*.340*	**1.39**	1.08–1.79	*.012*	**1.24**	1.00–1.55	*.055*
Any behavioural symptom	**0.90**	0.46–1.76	*.758*	**1.29**	0.98–1.7	*.066*	**1.49**	1.13–1.95	*.004*
Worsening of behavioural symptoms in 90 days	**2.32**	0.88–6.15	*.090*	**1.59**	1.10–2.30	*.014*	**1.45**	1.02–2.05	*.038*
Poor self-rated health	**2.15**	1.38–3.37	*.001*	**1.18**	0.96–1.46	*.122*	**1.43**	1.17–1.75	< *.001*
Weight loss^a^	Didn’t appear at level 1			**0.93**	0.56–1.56	*.790*	**1.83**	1.22–2.75	*.004*
Body mass index, kg/m2									
< 18.5	**1.12**	0.46–2.71	*.807*	**1.44**	0.87–2.40	*.154*	**0.78**	0.47–1.29	*.335*
18.5–23.9	**1**			**1**			**1**		
24–29.9	**0.84**	0.55–1.28	*.412*	**1.16**	0.92–1.47	*.210*	**1.05**	0.83–1.31	*.700*
≥ 30	**0.63**	0.36–1.12	*.114*	**0.98**	0.75–1.29	*.901*	**0.72**	0.54–0.94	*.018*
Pain Scale (0–3)									
0–1	**1**			**1**			**1**		
2–3	**1.11**	0.75–1.65	*.602*	**1.13**	0.93–1.37	*.224*	**1.29**	1.07–1.57	*.009*
**Diagnoses**^b^									
Congestive heart failure^a^	**1.33**	0.77–2.31	*.312*	**1.21**	0.97–1.51	*.089*	**1.26**	1.03–1.54	*.022*
Coronary artery disease^a^	**1.72**	1.13–2.62	*.011*	**1.01**	0.81–1.26	*.945*	**1.26**	1.03–1.54	*.022*
Parkinson's disease	**1.95**	0.61–6.21	*.259*	**1.58**	0.79–3.15	*.195*	**2.27**	1.10–4.67	*.027*
Musculoskeletal disorders	**1.1**	0.60–2.01	*.763*	**1.35**	1.01–1.82	*.045*	**1.18**	0.88–1.58	*.262*
Old other fracture	**5.56**	2.05–15.05	*.001*	**1.22**	0.73–2.02	*.449*	**1.30**	0.82–2.07	*.263*
History of urinary tract infection^a^	**3.86**	1.75–8.52	*.001*	**1.53**	0.89–2.61	*.121*	**2.25**	1.57–3.22	< *.001*
Renal insufficiency^a^	**1.06**	0.52–2.15	*.868*	**1.63**	1.11–2.39	*.013*	**1.18**	0.92–1.51	*.203*
**Medication**									
Psychotropic medication	**0.86**	0.59–1.24	*.406*	**1.16**	0.95–1.41	*.137*	**1.31**	1.07–1.59	*.008*
**Health stability**									
Changes in Health, End-Stage Disease, Signs, and Symptoms Scale score (0–5)									
0	**1**			**1**			**1**		
1	**1.14**	0.74–1.76	*.546*	**1.38**	1.07–1.77	*.012*	**1.53**	1.16–2.01	*.003*
2–5	**0.45**	0.06–3.50	*.445*	**1.51**	1.19–1.91	*.001*	**1.91**	1.49–2.45	< *.001*

^a^Included in the DIVERT scale

^b^All previously diagnosed diseases although they do not require monitoring or treatment at the time of assessment, and diagnoses as a reason for hospitalization in 90 days prior to assessment

### The accuracy of modified DIVERT Scale

Of the available determinants, impaired cognition (CPS > 2) and urinary incontinence were considered in the modification of DIVERT algorithm based on earlier literature [13, 43, 54] and as they were strongly associated with hospitalization in some but not all DIVERT groups. The AUC of both modified DIVERT scales was 0.64 (Table 4).

**Table 4:** Values of AUC for original (based on our earlier study using the same data [42]) and modified DIVERT algorithms in the whole data and in different age groups

**Age group**	**DIVERT**					
	**Original**		**Modified 1**		**Modified 2**	
	**AUC**	**95% CI**	**AUC**	**95% CI**	**AUC**	**95% CI**
**ALL**	**0.62**	0.60–0.64	**0.64**	0.63–0.65	**0.64**	0.62–0.65
** < 70**	**0.71**	0.65–0.77	**0.72**	0.66–0.78	**0.72**	0.66–0.78
**70–79**	**0.66**	0.62–0.69	**0.66**	0.63–0.70	**0.66**	0.63–0.69
**80–89**	**0.60**	0.58–0.62	**0.62**	0.60–0.64	**0.61**	0.59–0.64
** ≥ 90**	**0.59**	0.56–0.63	**0.61**	0.57–0.64	**0.60**	0.57–0.64

Distribution of the DIVERT scores, and absolute risk, sensitive and specificity, and odds ratio for unplanned hospitalization for the original and the modified DIVERT algorithms are presented in Additional files 3 and 4. There was a slight trend to better sensitivity when using modified DIVERT scale, especially in older age groups, but the specificity was the same or somewhat worse. More admissions occurred within the higher scores of modified DIVERT scale: in the original DIVERT scale there were 822 admissions (49.6%) at levels 4–6 and in the modified DIVERT scales the corresponding figures were 987 (59.5%) and 946 admissions (57%) (Additional file 3).

## Discussion

This study indicated that the incidence of hospitalization among the oldest home care clients was high and the most common specific reasons for hospitalization were infectious diseases, cardiovascular diseases, and dementia diseases, confirming the results of previous studies also for the oldest home care clients [43]. Like in the earlier studies [11, 13, 55], there were many geriatric symptoms and syndromes associated with hospital admissions but adding cognitive performance and urinary incontinence to the DIVERT algorithm did not improve the accuracy of the algorithm in classifying the risk for unplanned hospitalization but it remained at the same poor level as previously reported [41, 42, 56]. However, the low risk level classification meant lower absolute risk for hospitalization than in the original DIVERT.

Cardiovascular, cerebrovascular, and musculoskeletal diseases and a group of other specific diseases represented a clear majority of discharge diagnoses at high risk DIVERT levels 4–6. This is understandable because the drivers of DIVERT algorithm include information about cardiorespiratory symptoms, cardiac conditions, and diagnoses [39]. However, nearly half of infectious diseases and more than half of other main discharge diagnosis groups (dementia diseases, injuries and geriatric symptoms) were present at low or moderate risk levels of DIVERT groups, raising suspicion that the algorithm doesn’t identify the risk factors behind these diagnoses.

In an earlier study among home care clients [43], daily urinary incontinence, chronic skin ulcers, and both functional and cognitive impairment increased the likelihood that the reason for hospitalization was infectious diseases. In the present study, urinary incontinence was associated with hospitalizations both in the high risk (levels 4–6) and moderate risk (levels 2–3) DIVERT groups, and cognitive impairment in the moderate risk group (levels 2–3). Urinary incontinence and cognitive impairment are not included in the original DIVERT algorithm, whereas skin ulcers are. In addition to infectious diseases, an association between impaired cognitive capacity and hospitalizations due to dementia has been described [13, 43].

In earlier studies concerning risk prediction of hospitalization among old people, the most frequently incorporated predictors have been medical comorbidities, impairments in ADLs, age, quality of life and previous recent healthcare utilisation [31]. According to the review concerning risk prediction models developed for risk classifying of unplanned hospitalizations [31], assessment of cognition or other mental disorders, physical limitations, medication, and nutritional status were included in only less than half of the models, weight loss and previous falls in only two out of twelve models. Urinary incontinence as a predictive variable was not included in any predictive model mentioned.

These observations made us think that the risk for hospitalization of clients with these characteristics may be underestimated in the algorithm. Therefore, impaired cognitive function and urinary incontinence were included in the modified DIVERT algorithms, hoping they could improve the accuracy. The associations of these factors with hospitalization or infectious diseases have also been described in earlier studies among old adults [57–59]. Supplementing of the algorithm was also supported by the earlier observations suggesting that combined use of different scales may be more accurate than a single measure in identifying persons at risk for hospitalization [60], though the results are somewhat contradictory [61]*.* Although it is acknowledged that these two variables were excluded when building the original DIVERT algorithm [39], we considered our approach reasonable because exclusion of these two variables may be due to the technique used in variable selection and because age-dependent predictors were not specifically considered in the original study [39].

The results of this study support our original idea, that there are geriatric symptoms and syndromes not included in the DIVERT algorithm but associated with the low or moderate risk of hospitalization in the DIVERT that should be taken into account in clients’ care plan. Against expectations, however, modifying of algorithm didn’t improve its ability to classify the risk compared to the original version, and the AUCs of 0.62 for the original [42] and 0.64 for the modified versions represent poor accuracy. There was a slight trend to better sensitivity at the cost of worsening specificity, especially in older age groups.

When evaluating the uselfulness of screening tests, predictive values are more relevant than are sensitivity and specificity. Sensitivity is the ability of a test to find those with the condition, and at the same time to avoid false negatives, while specificity denotes the ability of a test to identify those without the condition and, at the same time, to avoid false positives. However, a highly sensitive test, though yielding a positive result, doesn’t indicate that a condition is present, and a highly specific test, when yielding a negative result, doesn’t indicate that a condition is absent. In a screening situation, sensitivity and specificity can be useful if they are very high. It is unlikely, that a highly sensitive screening test produces false negative outcomes and highly specific screening test false positive results [62].

In our study, better sensitivity and worsening specificity in the modified models means that more outcomes occur with higher scores but at the same time there are more false positives. However, an improvement in sensitivity, especially at the lower risk levels when using the modified models, may indicate that there are fewer false negatives, in other words, there are correctly more those without a real risk for hospitalization, than when using the original DIVERT. Consequently, the absolute number of hospital admissions decreased after modifications especially at the low risk level 2.

There are several explanations for why the accuracy of the algorithm did not improve. Geriatric symptoms and syndromes present in the DIVERT are already wide-ranging. On the other hand, clients’ medical, functional, or social situation may change after the MDS-HC assessment, creating new combinations of variables related to the individual’s risk of hospitalization, impacting the overall risk, and reducing the importance of single risk factors identified earlier in the assessment. Also the facts that different factors predispose to hospitalization for different reasons [43] and the reasons for hospitalization may be manifold and vary from time to time, can explain why risk classification models containing only a few variables probably have limited accuracy. Also, the older the person is, the more common diseases and geriatric syndromes are [63, 64], and therefore the possibility to predict risk based on a limited set of variables may be reduced. This is highlighted in the frailest group of home care clients, as per its definition, frailty is a measure of vulnerability and in frail older adults even minor health issues may lead to unplanned hospitalizations. Frailty is also often associated with other geriatric syndromes and according to the accumulated deficit model, frailty is a result of multiple underlying health issues [65]. Although MDS-HC can be used to calculate frailty index [66, 67], we examined associations between single variables (e.g. different symptoms, syndromes and chronic conditions that could also underlie or be markers of frailty) and hospitalization. Another reason for not using frailty index is that its idea is to present frailty as a continuum while our algorithm approach requires a dichotomous variable. Earlier, instruments and scores derived from the accumulated deficit model of frailty, e.g. the Rockwood’s Frailty Index [68] and the Clinical Frailty Scale [69], have been shown to predict the risk of death as well as re-hospitalization, extended length of stay, and institutionalisation [34, 35]. However, the usefulness of frailty scales alone in classifying the risk of unplanned hospital admissions is unclear, and their AUCs (0.59–0.63) are unconvincing [38]. From clinical point of view, recognition of frailty among patients at high risk of hospitalization, based on DIVERT, is still important because of its high association with several adverse outcomes. Indeed, it has been suggested that CGA could be used in such situations to identify frailty and use this information to improve overall care of frail older adults [70].

The present results raise the question how meaningful attempting to classify an individual home care client’s risk for hospitalization really is, as such persons are at a complex clinical context, due to their multimorbidity, health instability and reduced functional capacity, and have many kinds of individual characteristics predicting different adverse outcomes, not only hospitalization [71, 72]. If screening models are used, models designed and validated to predict multiple or composite outcomes might be more useful as the commonly known risk factors may have different consequences at the level of an individual [73].

Despite these concerns and problems with the accuracy of DIVERT – from the physician’s point of view – the automatically available risk classification of DIVERT scale together with the other results of the MDS-HC assessments support decision-making in care planning and identifying those in need of the most urgent interventions and more frequent follow-up. The present results concerning the MDS-HC variables associated with hospitalization at different DIVERT levels (Table 5) broaden understanding which issues in different clients’ health profile appear as potential targets for interventions, such as CGA, and should be considered in care plans aiming to prevent hospitalizations. At least some of the risk factors can be affected and/or treated. Particular attention should be paid to discovering the reasons for the worsening of ADL or cognitive function, unstable health situation, falls and unsteady gait, urinary incontinence, and weight loss. Whether there are some DIVERT-level-specific interventions that take into account the associations found in this study between DIVERT levels and client characteristics, is a subject of further research.

**Table 5:** Variables included in the DIVERT scale, and MDS-HC variables (not included in DIVERT) associated with hospitalization at some DIVERT group level in the follow up of 180 days

DIVERT level	DIVERT variables	DIVERT groups	Variables associated with hospitalization
DIVERT 1	• No previous ED or hospital visits or other DIVERT variables	1	• Age ≥ 90 years • Faecal incontinency weekly • Activities of Daily Living Hierarchy score 1–2 • Unstable walking • *Poor self-rated health*^*a*^ • *Old other fracture*^*a*^
DIVERT 2	• Cardiorespiratory symptoms • Falls • Stasis ulcers • ADL decline • Problems in nutrition	2–3	• Age ≥ 90 years^a^ • *Client doesn't believe he/she is capable of improving performance in physical function*^*a*^ • *Activities of Daily Living Hierarchy score 1–2*^*a*^ • *Cognitive Performance Scale score 1–6*^*a*^ • Impairment of decision-making capacity • *Worsening of social interaction*^*a*^ • *Urinary incontinence daily*^*a*^ • *Worsening of urinary incontinence*^*a*^ • *Unstable walking*^*a*^ • *Fear of walking*^*a*^ • *Chronic skin ulcers*^*a*^ • *Changes in Health, End-Stage Disease, Signs, and Symptoms Scale score* ≥ *2*^*a*^
DIVERT 3	• Previous ED or hospital visit (1) ◦ and poor prospects for functional improvement ◦ or any cardiac condition • Cardio-respiratory symptoms ◦ and problems in nutrition • Falls ◦ and diagnosis of stroke or diabetes		
DIVERT 4	• Previous hospital or ED use (1) ◦ and cardiorespiratory symptoms ◦ or poor prospects for functional improvement and mood symptoms • Cardio-respiratory symptoms ◦ and any cardiac condition and diagnosis of COPD/renal failure / UTI / pneumonia ◦ or oxygen therapy	4–6	• *Age* ≥ *90 years*^*a*^ • Client doesn't believe he/she is capable of improving performance in physical function • Activities of Daily Living Hierarchy score 3–4 • *Decreased social interaction* • *Urinary incontinence daily*^*a*^ • Unstable walking • Fear walking • *Chronic skin ulcers*^*a*^ • Any behavioural symptom • *Poor self-rated health*^*a*^ • Pain Scale 2–3 • Psychotropic medication • *Changes in Health, End-Stage Disease, Signs, and Symptoms Scale score* ≥ *1*^*a*^
DIVERT 5	• Hospital or ED visits (≥ 2) • ED or hospital visit (1) ◦ and cardiorespiratory symptoms and any cardiac condition		
DIVERT 6	• Hospital or ED visits (≥ 2) ◦ and cardiorespiratory symptoms ◦ and urinary catheter or UTI		

^a^*p* ≤ .001 *COPD* Chronic Obstructive Pulmonary Disease *UTI* Urinary Tract Infection

Moreover, these results could be utilized in building a new Clinical Assessment Protocol (CAP), an algorithm produced by the MDS-HC to prompt health care professionals about client’s possible problems, risk factors or potential for improved function that may require clinical intervention and should be taken into account in the care plan [74, 75]. A CAP including the findings of this study could be one way to bring these conditions to the attention and help physicians and other professionals to understand better the patient's situation, needs in care and risk of adverse outcomes, together with the information of the DIVERT scale.

Our study has some limitations, mainly concerning the data. The research is based on MDS-HC data and discharge records from a single city. The regional differences in types and availability of services, such as supply of hospital care beds, existing resources in the primary care, and different home care practices may affect hospital utilization rates, risk of hospitalization, and risk prediction limiting its generalizability to rural areas and other countries. On the other hand, access to health care should not bias our findings, thanks to the publicly funded health care services in Finland. Although the present study was based on the data collected in 2014 and 2015, the organization and coverage of home care in the studied area have remained essentially unchanged whereas the use of hospital care in general in the whole country has reduced [76]. However, it is unlikely that the predictors of hospitalizations would have changed as most hospitalizations are related to acute diseases. One such effort has been an attempt to treat clients’ acute situations at their homes with intensified home care and home hospital services. However, our data does not allow us to clarify this in more detail. According to national guidelines, a new MDS-HC assessment should be performed when there is a significant change in the client’s health condition, but it is unclear how well this has really occurred. If the health status has changed, the last assessment doesn’t reflect the clients’ real condition. The reliability of the assessments could not be assessed on the basis of the data. However, in the City of Tampere, nurses’ competence for carrying out the assessments has been ensured e.g. by having a RAI online course, exam, and exercise assessment for new employees. Statistical strength may have suffered in some groupings of data, leading to small amounts of single variables distorting conclusions. Due to the lack of clinical data, the reliability of individual diagnoses couldn’t be confirmed and only the main diagnoses were registered although there may have been also other reasons for hospital care. The limitations in the diagnosis data should, however, not bias comparisons between different DIVERT groups.

When the number of events is low relative to the number of predictors, standard regression analysis may produce overfitted models that tend to underestimate the probability of an event in low-risk patients and overestimate it in high-risk patients [77]. This comes true with some variables in our study when there are less events per variable (EPV) than the 10–15 EPV that has been recommended [78] (Table 1). However, these results appeared clinically relevant and reasonable.

On the other hand, the strength of the data is that it represents well typical home care clients in an urban area and the covered ca. 85% of home care clients in the area. Moreover, the aim to investigate the risk for hospitalization of the oldest clients was well achieved as 23.5% of the assessments and 29% of the admissions were among those of aged 90 years or older. The discharge records were practically complete, thanks to obligatory electronic recording of hospital discharges to the nationwide Finnish Hospital Discharge Register that covers > 95% of discharges whose completeness and accuracy is from satisfactory to very good [79]. The types and availability of services were the same in the whole area and they had no effect on hospital admission rates.

## Conclusion

The geriatric challenges and diagnoses associated with unplanned hospitalization partly differ between home care clients with low, moderate, and high risk of hospitalization, according to the DIVERT scale. Taking cognitive impairment and urinary incontinence into account, however, did not improve the accuracy of the DIVERT scale despite better sensitivity. In home care clients’ complex clinical context more important than a risk score based on a limited number of risk factors may be the individual different combinations of risk factors and their interactions. Nevertheless, DIVERT as a score, produced using routinely collected MDS-HC data, could be used to target geriatric services especially to those home care clients who might benefit most of a CGA-based individual prevention, treatment and rehabilitation plan. Whether DIVERT or similar risk scales could be used to improve effectiveness and efficacy of geriatric services and interventions in home care warrant further research.

### Supplementary Information

**Additional file 1.** The diagnosis groups (grouped according to the first registered diagnosis, and respective diagnosis codes according to the 10^th^ revision of the International Classification Diseases). 

**Additional file 2.** The univariate analysis showing the associations between MDS-HC determinants and hospitalization during the follow up of 180 days from the index MDS-HC assessment.

**Additional file 3.** Distribution of DIVERT scores, absolute risk, sensitivity and specificity, and odds ratio of unplanned hospitalization, according to DIVERT score including original  (based on our earlier study using the same data ^18^) and modified DIVERT algorithms.

**Additional file 4.** Sensitivity and specificity of the original (based on our earlier study using the same data ^18^)  and modified DIVERT scales in the whole data and in different age groups.

## Data Availability

The datasets used and/or analysed during the current study are available from the corresponding author on reasonable request.

## Abbreviations

ADLh: Activities of daily living performance scale
AUC: Area under the curve
BMI: Body mass index
CAPs: Clinical assessment protocols
CGA: Comprehensive geriatric assessment
CHESS: Changes in health, end-stage diseases, signs, and symptoms scale
CI: Confidence interval
CPS: Cognitive performance scale
DIVERT: Detection of indicators and vulnerabilities for emergency room trips scale
DRS: Depression scale
ED: Emergency department
EPV: Events per variable
interRAI-HC: InterRAI-home care
MDS-HC: Minimum data set for home care
OR: Odds ratio
PAIN: Pain scale
ROC: Receiver operating characteristic curve
