# Supplementary material for: The role of geriatric syndromes in predicting unplanned hospitalizations: a population-based study using Minimum Data Set for Home Care
Source: BMC Geriatr. 2023 Oct 26;23:696. doi: 10.1186/s12877-023-04408-w (PMC10605458; doi:10.1186/s12877-023-04408-w)
Supplement: Supplementary file 1 — Additional file 1. The diagnosis groups (grouped according to the first registered diagnosis, and respective diagnosis codes according to the 10th revision of the International Classification Diseases). [file 12877_2023_4408_MOESM1_ESM.docx]

| **Additional file 1.** **The diagnosis groups (grouped according to the first registered diagnosis, and respective diagnosis codes according to the 10^th^ revision of the International Classification Diseases).** | | | | | | |  |  |
| --- | --- | --- | --- | --- | --- | --- | --- | --- |
|  |  |  |  |  |  |  |  |  |
| **Group 1: Infectious diseases** | | |  |  |  |  |  |  |
| A00–B99 Infectious and parasitic diseases | | | | |  |  |  |  |
| J00–J06 Acute upper respiratory infections | | | | |  |  |  |  |
| J10–J18 Influenza and pneumonia | | | |  |  |  |  |  |
| J20–J22 Other acute lower respiratory infections | | | | |  |  |  |  |
| Urinary tract infections | |  |  |  |  |  |  |  |
|  | N10 Acute tubulo-interstitial nephritis | | | |  |  |  |  |
|  | N30 Cystitis | |  |  |  |  |  |  |
|  | N39.0 Urinary tract infection, site not specified | | | | |  |  |  |
|  |  |  |  |  |  |  |  |  |
| **Group 2: Dementia diseases** | | |  |  |  |  |  |  |
| F00-F03 Dementia | |  |  |  |  |  |  |  |
| G30–G32 Other degenerative diseases of the nervous system | | | | | |  |  |  |
|  |  |  |  |  |  |  |  |  |
| **Group 3: Cardiovascular diseases** | | | |  |  |  |  |  |
| I10 Hypertension | |  |  |  |  |  |  |  |
| I11 Hypertensive heart diseases | | |  |  |  |  |  |  |
| I20–I25 Ischaemic heart diseases | | | |  |  |  |  |  |
| I34-I37 Valve disorders | |  |  |  |  |  |  |  |
| I42 Cardiomyopathy | |  |  |  |  |  |  |  |
| I47-I49 Cardiac arrhythmias | | |  |  |  |  |  |  |
| I50 Heart failure | |  |  |  |  |  |  |  |
| I71 Aortic aneurysm and dissection | | | |  |  |  |  |  |
| I72 Other aneurysm and dissection | | | |  |  |  |  |  |
|  |  |  |  |  |  |  |  |  |
| **Group 4: Cerebrovascular diseases** | | | |  |  |  |  |  |
| I60–I69 Cerebrovascular diseases | | | |  |  |  |  |  |
| G45 Transient cerebral ischaemic attacks and related syndromes | | | | | | |  |  |
|  |  |  |  |  |  |  |  |  |
| **Group 5: Musculoskeletal diseases** | | | |  |  |  |  |  |
| M00–M99 Diseases of the musculoskeletal system and connective tissue | | | | | | |  |  |
|  |  |  |  |  |  |  |  |  |
| **Group 6: Injuries** | |  |  |  |  |  |  |  |
| S00–S09 Injuries to the head | | |  |  |  |  |  |  |
| S10–S19 Injuries to the neck | | |  |  |  |  |  |  |
| S20–S29 Injuries to the thorax | | |  |  |  |  |  |  |
| S30–S39 Injuries to the abdomen, lower back, lumbar spine, and pelvis | | | | | | |  |  |
| S40–S49 Injuries to the shoulder and upper arm | | | | |  |  |  |  |
| S50–S59 Injuries to the elbow and forearm | | | | |  |  |  |  |
| S60-S69 Injuries to the wrist and hand | | | |  |  |  |  |  |
| S70–S79 Injuries to the hip and thigh | | | |  |  |  |  |  |
| S80–S89 Injuries to the knee and lower leg | | | | |  |  |  |  |
| S90–S99 Injuries to the ankle and foot | | | |  |  |  |  |  |
| T20-T32 Burns and corrosions | | |  |  |  |  |  |  |
| T36-T50 Poisoning by drugs, medicaments, and biological substances | | | | | | |  |  |
|  |  |  |  |  |  |  |  |  |
| **Group 7: Other specific diseases** | | | |  |  |  |  |  |
| Malignant neoplasms | |  |  |  |  |  |  |  |
|  | C00–C14 Malignant neoplasms of lip, oral cavity, and pharynx | | | | | |  |  |
|  | C15–C26 Malignant neoplasms of digestive organs | | | | | |  |  |
|  | C30–C39 Malignant neoplasms of respiratory and intrathoracic organs | | | | | | |  |
|  | C43–C44 Melanoma and other malignant neoplasms of skin | | | | | |  |  |
|  | C45–C49 Malignant neoplasms of mesothelial and soft tissue | | | | | |  |  |
|  | C50–C50 Malignant neoplasm of breast | | | | |  |  |  |
|  | C51–C58 Malignant neoplasms of female genital organs | | | | | |  |  |
|  | C60–C63 Malignant neoplasms of male genital organs | | | | | |  |  |
|  | C64–C68 Malignant neoplasms of urinary tract | | | | |  |  |  |
|  | C69-C97 Other malignant neoplasms | | | |  |  |  |  |
|  | D00-D09 In situ neoplasms | | |  |  |  |  |  |
| Benign neoplasms | |  |  |  |  |  |  |  |
|  | D10-D36 Benign neoplasms | | | |  |  |  |  |
| Diseases of the blood and blood-forming organs | | | | |  |  |  |  |
|  | D50-D53 Nutritional anaemias | | | |  |  |  |  |
|  | D60-D64 Aplastic and other anaemias | | | |  |  |  |  |
| Endocrine, nutritional, and metabolic diseases | | | | |  |  |  |  |
|  | E00-E07 Disorders of thyroid gland | | | |  |  |  |  |
|  | E10-E14 Diabetes mellitus | | |  |  |  |  |  |
| Organic, including symptomatic, mental disorders | | | | |  |  |  |  |
|  | F20 Schizophrenia | |  |  |  |  |  |  |
|  | F22 Persistent delusional disorders | | | |  |  |  |  |
|  | F25 Schizoaffective disorders | | | |  |  |  |  |
|  | F30-F31 Bipolar affective disorder | | | |  |  |  |  |
|  | F32-F33 Depressive disorder | | | |  |  |  |  |
| Diseases of the nervous system | | |  |  |  |  |  |  |
|  | G20 Parkinson disease | | |  |  |  |  |  |
|  | G21Secondary parkinsonism | | |  |  |  |  |  |
|  | G40 Epilepsy | |  |  |  |  |  |  |
| Diseases of the circulatory system | | | |  |  |  |  |  |
|  | I26 Pulmonary embolism | | |  |  |  |  |  |
|  | I70 Atherosclerosis | |  |  |  |  |  |  |
|  | I80 Phlebitis and thrombophlebitis | | | |  |  |  |  |
| Diseases of the respiratory system | | | |  |  |  |  |  |
|  | J40–J47 Chronic lower respiratory diseases | | | | |  |  |  |
| Diseases of the digestive system | | | |  |  |  |  |  |
|  | K20–K31 Diseases of oesophagus, stomach, and duodenum | | | | | |  |  |
|  | K57 Diverticular disease of intestine | | | |  |  |  |  |
|  | K70-K77 Diseases of liver | | |  |  |  |  |  |
|  | K80 Cholelithiasis | |  |  |  |  |  |  |
|  | K81-K87 Disorders of gallbladder, biliary tract, and pancreas | | | | | |  |  |
| Diseases of the genitourinary system | | | |  |  |  |  |  |
|  | N17–N19 Renal failure | | |  |  |  |  |  |
|  | N40-N51 Diseases of male genital organs | | | | |  |  |  |
|  |  |  |  |  |  |  |  |  |
| **Group 8: Geriatric symptoms** | | |  |  |  |  |  |  |
| E86-E87 Volume depletion and other disorders of fluid, electrolyte, and acid-base balance | | | | | | | |  |
| E40-E46 Malnutrition | |  |  |  |  |  |  |  |
| E63 Other nutritional deficiencies | | | |  |  |  |  |  |
| F05.1 Delirium superimposed on dementia | | | |  |  |  |  |  |
| F05.0Delirium not superimposed on dementia | | | | |  |  |  |  |
| F05.8-F0.59 Other or unspecified delirium | | | |  |  |  |  |  |
| I95 Idiopathic hypotension | | |  |  |  |  |  |  |
| N39.3 Stress incontinence | | |  |  |  |  |  |  |
| R06.0 Dyspnoea | |  |  |  |  |  |  |  |
| R41 Other symptoms and signs involving cognitive functions and awareness | | | | | | |  |  |
| R42 Dizziness and giddiness | | |  |  |  |  |  |  |
| R44 Other symptoms and signs involving general sensations and perceptions | | | | | | | |  |
| R53 Malaise and fatigue | | |  |  |  |  |  |  |
| R55 Syncope and collapse | | |  |  |  |  |  |  |
| G47 Sleep disorders | |  |  |  |  |  |  |  |
| R52 Pain, not elsewhere classified | | | |  |  |  |  |  |
|  |  |  |  |  |  |  |  |  |
| **Group 9: Other diseases and symptoms** | | | | |  |  |  |  |
| D37-D48 Neoplasms of uncertain or unknown behaviour | | | | | |  |  |  |
| E15-E16 Other disorders of glucose regulation and pancreatic internal secretion | | | | | | | |  |
| E66 LIHAVUUS | |  |  |  |  |  |  |  |
| E83 Disorders of mineral metabolism | | | |  |  |  |  |  |
| F06Other mental disorders due to brain damage and dysfunction and to physical disease | | | | | | |  |  |
|  |  |  |  |  |  |  |  |  |
| F10-F19 Mental and behavioural disorders due to psychoactive substance use, excluding F10.73 alcoholic dementia | | | | | | | |  |
|  |  |  |  |  |  |  |  |  |
| F23 Brief psychotic disorders | | |  |  |  |  |  |  |
| F29Unspecified nonorganic psychosis | | | |  |  |  |  |  |
| F34.9Persistent mood [affective] disorder, unspecified | | | | | |  |  |  |
| F41 Other anxiety disorders | | |  |  |  |  |  |  |
| F60 Specific personality disorders | | | |  |  |  |  |  |
| F80 Specific developmental disorders of speech and language | | | | | |  |  |  |
| G10-G14Systemic atrophies primarily affecting the central nervous system | | | | | | |  |  |
| G25 Other extrapyramidal and movement disorders | | | | |  |  |  |  |
| G50-G59 Nerve, nerve root and plexus disorders | | | | |  |  |  |  |
| G70-G73 Diseases of myoneural junction and muscle | | | | | |  |  |  |
| G80-G83 Cerebral palsy and other paralytic syndromes | | | | | |  |  |  |
| G89-G99 Other disorders of the nervous system | | | | |  |  |  |  |
| H00-H59 Diseases of the eye and adnexa | | | |  |  |  |  |  |
| H60-H95 Diseases of the ear and mastoid process | | | | |  |  |  |  |
| I44 Atrioventricular and left bundle-branch block | | | | |  |  |  |  |
| I46 Cardiac arrest | |  |  |  |  |  |  |  |
| I82-I87 Disorders of veins | | |  |  |  |  |  |  |
| J60-J70 Lung diseases due to external agents | | | | |  |  |  |  |
| J80-J84 Other respiratory diseases principally affecting the interstitium | | | | | | |  |  |
| J85-J86 Suppurative and necrotic conditions of the lower respiratory tract | | | | | | |  |  |
| J90-J94 Other diseases of the pleura | | | |  |  |  |  |  |
| J96 Respiratory failure, not elsewhere classified | | | | |  |  |  |  |
| J98 Other respiratory disorders | | |  |  |  |  |  |  |
| K00-K14 Diseases of oral cavity, salivary glands, and jaws | | | | | |  |  |  |
| K40-K46 Hernia | |  |  |  |  |  |  |  |
| K50-K52 Noninfective enteritis and colitis | | | |  |  |  |  |  |
| K55 Vascular disorders of intestine | | | |  |  |  |  |  |
| K56 Paralytic ileus and intestinal obstruction without hernia | | | | | |  |  |  |
| K58-K59 Irritable bowel syndrome or other functional intestinal disorders | | | | | | |  |  |
| K61 Abscess of anal and rectal regions | | | |  |  |  |  |  |
| K62 Other diseases of anus and rectum | | | |  |  |  |  |  |
| K90-K93 Other diseases of the digestive system | | | | |  |  |  |  |
| L00-L99 Diseases of the skin and subcutaneous tissue | | | | | |  |  |  |
| N00-N08 Glomerular diseases | | |  |  |  |  |  |  |
| N12 Tubulo-interstitial nephritis, not specified as acute or chronic | | | | | | |  |  |
| N31 Neuromuscular dysfunction of bladder, not elsewhere classified | | | | | | |  |  |
| N32 Other disorders of bladder | | |  |  |  |  |  |  |
| N35 Urethral stricture | |  |  |  |  |  |  |  |
| N80-N98 Noninflammatory disorders of female genital tract | | | | | |  |  |  |
| R02 Gangrene, not elsewhere classified | | | |  |  |  |  |  |
| R05 Cough |  |  |  |  |  |  |  |  |
| R07 Pain in throat and chest | | |  |  |  |  |  |  |
| R09 Other symptoms and signs involving the circulatory and respiratory systems | | | | | | | |  |
| R10 Abdominal and pelvic pain | | |  |  |  |  |  |  |
| R11 Nausea and vomiting | | |  |  |  |  |  |  |
| R25-R29 Symptoms and signs involving the nervous and musculoskeletal systems | | | | | | | |  |
| R31 Unspecified haematuria | | |  |  |  |  |  |  |
| R33 Retention of urine | | |  |  |  |  |  |  |
| R35 Polyuria | |  |  |  |  |  |  |  |
| R40 Somnolence, stupor, and coma | | | |  |  |  |  |  |
| R47-R49 Symptoms and signs involving speech and voice | | | | | |  |  |  |
| R50 Fever of other and unknown origin | | | |  |  |  |  |  |
| R56 Convulsions, not elsewhere classified | | | | |  |  |  |  |
| R60 Oedema, not elsewhere classified | | | |  |  |  |  |  |
| R91Abnormal findings on diagnostic imaging of lung | | | | |  |  |  |  |
| T80-T88 Complications of surgical and medical care, not elsewhere classified excluding T81.4 infection following a procedure, not elsewhere classified  and T84.5 infection and inflammatory reaction due to internal joint prosthesis | | | | | | | |  |
| Z00-Z99 Factors influencing health status and contact with health services | | | | | | |  |  |
