# Supplementary material for: The role of geriatric syndromes in predicting unplanned hospitalizations: a population-based study using Minimum Data Set for Home Care
Source: BMC Geriatr. 2023 Oct 26;23:696. doi: 10.1186/s12877-023-04408-w (PMC10605458; doi:10.1186/s12877-023-04408-w)
Supplement: Supplementary file 2 — Additional file 2. The univariate analysis showing the associations between MDS-HC determinants and hospitalization during the follow up of 180 days from the index MDS-HC assessment. [file 12877_2023_4408_MOESM2_ESM.docx]

| **Additional file 2. The univariate analysis showing the associations between MDS-HC determinants and hospitalization during the follow up of 180 days from the index MDS-HC assessment** | | | |  |
| --- | --- | --- | --- | --- |
|  |  |  |  |  |
|  |  |  |  |  |
|  |  |  |  |  |
|  | **Univariate** | |  |  |
|  | **OR** | **95% CI** | ***p*** |  |
|  |  |  |  |  |
| **Demographic** |  |  |  |  |
| Age |  |  |  |  |
| 80-89 | **1** |  |  |  |
| 90+ | **1.5** | 1.30-1.73 | <.001 |  |
| Male | **1** |  |  |  |
| Female | **0.933** | 0.80-1.09 | .368 |  |
| **Social situation** |  |  |  |  |
| Caregiver stressed | **1.51** | 1.16-1.97 | .002 |  |
| Housing-related problems | **1.29** | 1.12-1.49 | <.001 |  |
| Use or needs of services |  |  |  |  |
| Reason for home care: client has been discharged from hospital | **1.27** | 1.11-1.44 | <.001 |  |
| Acute outpatient care or unplanned hospitalization in 90 days before assessment ^a^ | **1.77** | 1.56-2.01 | <.001 |  |
| **Function** |  |  |  |  |
| Activities of Daily Living Hierarchy score (0 - 6) |  |  |  |  |
| 0 | **1** |  |  |  |
| 1-2 | **2.18** | 1.65-2.87 | <.001 |  |
| 3-4 | **1.68** | 1.12-2.54 | .013 |  |
| 5-6 | **1.96** | 1.19-3.21 | .008 |  |
| ADL decline in previous 90 days ^a^ | **1.72** | 1.51-1.96 | <.001 |  |
| Poor prospects for functional improvement ^a^ | **1.13** | 0.89-1.43 | .324 |  |
| Client doesn't believe he/she is capable of improving performance in physical function | **1.3** | 1.14-1.49 | <.001 |  |
| Cognitive Performance Scale score (0-6) |  |  |  |  |
| 0 | **1** |  |  |  |
| 1-2 | **1.24** | 1.07-1.44 | .006 |  |
| 3-4 | **1.64** | 1.29-2.09 | <.001 |  |
| 5-6 | **2.21** | 1.57-3.10 | <.001 |  |
| Impairment of decision-making capacity | **1.45** | 1.27-1.67 | <.001 |  |
| Decreased social interaction | **1.45** | 1.23-1.72 | <.001 |  |
| Worsening of social interaction | **1.36** | 1.19-1.55 | <.001 |  |
| **Clinical symptoms** |  |  |  |  |
| Any cardio respiratory symptoms ^a^ | **1.45** | 1.27-1.65 | <.001 |  |
| Urinary incontinence daily | **1.55** | 1.35-1.78 | <.001 |  |
| Worsening of urinary incontinence | **1.47** | 1.26-1.71 | <.001 |  |
| Urinary catheter ^a^ | **1.12** | 0.64-1.97 | .684 |  |
| Faecal incontinency weekly | **1.36** | 1.06-1.74 | .015 |  |
| Chronic skin ulcers | **2.13** | 1.66-2.74 | <.001 |  |
| Stasis ulcer ^a^ | **2.33** | 1.71-3.19 | <.001 |  |
| Mouth problems | **1.3** | 1.12-1.51 | .001 |  |
| Vision |  |  |  |  |
| good enough | **1** |  |  |  |
| moderately impaired | **1.16** | 1.01-1.34 | .035 |  |
| severely impaired | **1.29** | 0.93-1.79 | .124 |  |
| Falls during 90 days before assessment ^a^ | **1.45** | 1.26-1.67 | <.001 |  |
| Unstable walking | **1.71** | 1.45-2.00 | <.001 |  |
| Fear of walking | **1.49** | 1.31-1.70 | <.001 |  |
| Depression Rating Scale score 0-14) |  |  |  |  |
| 0-2 | **1** |  |  |  |
| 3-14 | **1.52** | 1.29-1.80 | <.001 |  |
| Any mood symptoms ^a^ | **1.51** | 1.33-1.71 | <.001 |  |
| Increased mood symptoms in 90 days | **1.52** | 1.30-1.78 | <.001 |  |
| Any behavioural symptom | **1.39** | 1.16-1.66 | <.001 |  |
| Worsening of behavioural symptoms in 90 days | **1.7** | 1.33-2.17 | <.001 |  |
| Alcohol abuse | **0.64** | 0.39-1.06 | .085 |  |
| Feeling lonely | **1.01** | 0.88-1.16 | .909 |  |
| Poor self-reported health | **1.53** | 1.34-1.75 | <.001 |  |
| Weight loss ^a^ | **1.62** | 1.19-1.20 | .002 |  |
| Body mass index, kg/m2 |  |  |  |  |
| <18.5 | **1.07** | 0.77-1.48 | .709 |  |
| 18.5-23.9 | **1** |  |  |  |
| 24-29.9 | **1.04** | 0.89-1.20 | .646 |  |
| ≥ 30 | **0.81** | 0.68-0.97 | .022 |  |
| Decrease in food or fluids ^a^ | **1.37** | 1.01-1.85 | .044 |  |
| Pain Scale (0-3) |  |  |  |  |
| 0-1 | **1** |  |  |  |
| 2-3 | **1.31** | 1.16-1.49 | <.001 |  |
| **Diagnoses** |  |  |  |  |
| Congestive heart failure ^a^ | **1.45** | 1.26-1.67 | <.001 |  |
| Coronary artery disease ^a^ | **1.32** | 1.15-1.51 | <.001 |  |
| Alzheimer's disease | **0.91** | 0.80-1.04 | .180 |  |
| Other dementia | **1.14** | 0.94-1.39 | .197 |  |
| History of stroke ^a^ | **0.99** | 0.75-1.31 | .987 |  |
| Parkinson's disease | **1.84** | 1.18-2.89 | .007 |  |
| Musculoskeletal disorders | **1.28** | 1.06-1.56 | .011 |  |
| Old hip fracture | **1.47** | 1.05-2.06 | .024 |  |
| Old other fracture | **1.56** | 1.13-2.14 | .007 |  |
| Psychiatric diagnosis | **1.02** | 0.86-1.21 | .826 |  |
| Chronic obstructive pulmonary disease ^a^ | **1.24** | 1.02-1.51 | .031 |  |
| Cancer | **1.26** | 1.00-1.57 | .046 |  |
| Diabetes ^a^ | **1.12** | 0.97-1.28 | .113 |  |
| Pneumonia ^a^ | **1.60** | 0.88-2.90 | .121 |  |
| History of urinary tract infection ^a^ | **2.42** | 1.84-3.18 | <.001 |  |
| Renal insufficiency ^a^ | **1.52** | 1.25-1.84 | <.001 |  |
| **Medication** |  |  |  |  |
| Number of drugs ^b^ |  |  |  |  |
| 0-4 | **1** |  |  |  |
| 5-8 | **1.18** | 0.89-1.57 | .238 |  |
| 9 or more | **1.66** | 1.27-2.16 | <.001 |  |
| Psychotropic medication | **1.22** | 1.07-1.38 | .002 |  |
| Influenza vaccination | **0.98** | 0.86-1.11 | .696 |  |
| **Special therapies** |  |  |  |  |
| Oxygen therapy ^a^ | **2.04** | 1.12-3.49 | *.008* |  |
| **Health stability** |  |  |  |  |
| Changes in Health, End-Stage Disease, Signs, and Symptoms Scale score (0-5) |  |  |  |  |
| 0 | **1** |  |  |  |
| 1 | **1.53** | 1.30-1.80 | <.001 |  |
| 2-5 | **1.97** | 1.69-2.30 | <.001 |  |
| ^a^ included in the DIVERT scale |  |  |  |  |
| ^b^ including prescription and non-prescription medications | |  |  |  |
