# Supplementary material for: The role of geriatric syndromes in predicting unplanned hospitalizations: a population-based study using Minimum Data Set for Home Care
Source: BMC Geriatr. 2023 Oct 26;23:696. doi: 10.1186/s12877-023-04408-w (PMC10605458; doi:10.1186/s12877-023-04408-w)
Supplement: Supplementary file 3 — Additional file 3. Distribution of DIVERT scores, absolute risk, sensitivity and specificity, and odds ratio of unplanned hospitalization, according to DIVERT score including original (based on our earlier study using the same data 18) and modified DIVERT algorithms. [file 12877_2023_4408_MOESM3_ESM.docx]

| **Additional file 3. Distribution of DIVERT scores, absolute risk, sensitivity and specificity, and odds ratio of unplanned hospitalization, according to DIVERT score including original (based on our earlier study using the same data** ^18^**) and modified DIVERT algorithms.** | | | | | | | | |  |
| --- | --- | --- | --- | --- | --- | --- | --- | --- | --- |
|  |  |  |  |  |  |  |  |  |  |
|  |  |  |  |  |  |  |  |  |  |
|  |  |  |  |  |  |  |  |  |  |
|  |  |  |  |  |  |  |  |  |  |
| **DIVERT Score** | **Original DIVERT** | | | | | | | |  |
|  |  |  |  |  |  |  |  |  |  |
|  | **Number of assessments** | | **Number of outcomes** | |  |  |  |  |  |
|  |  |  |  |  | **Sensitivity** | **Specificity** | **OR** | **95%CI** |  |
|  | **N** | **%** | **N** | **%** |  |  |  |  |  |
| **1** | 1591 | 20.5 | 174 | 10.9 |  |  | 1 |  |  |
| **2** | 1992 | 25.7 | 364 | 18.3 | 0.90 | 0.23 | 1.82 | 1.50-2.21 |  |
| **3** | 1437 | 16.6 | 298 | 20.7 | 0.68 | 0.50 | 2.13 | 1.74-2.61 |  |
| **4** | 1166 | 15.1 | 320 | 27.4 | 0.50 | 0.69 | 3.08 | 2.51-3.78 |  |
| **5** | 894 | 11.5 | 258 | 28.9 | 0.30 | 0.83 | 3.30 | 2.67-4.09 |  |
| **6** | 664 | 8.6 | 244 | 36.7 | 0.15 | 0.93 | 4.73 | 3.79-5.91 |  |
| **Total** | 7744 | 100 | 1658 | 21.4 |  |  |  |  |  |
|  |  |  |  |  |  |  |  |  |  |
|  | **Modified DIVERT 1** | | | | | | | |  |
|  |  |  |  |  |  |  |  |  |  |
|  | **Number of assessments** | | **Number of outcomes** | |  |  |  |  |  |
|  |  |  |  |  | **Sensitivity** | **Specificity** | **OR** | **95%CI** |  |
|  | **N** | **%** | **N** | **%** |  |  |  |  |  |
| **1** | 1591 | 20.5 | 174 | 10.9 |  |  | 1 |  |  |
| **2** | 1280 | 16.5 | 199 | 15.5 | 0.9 | 0.23 | 1.50 | 1.21-1.87 |  |
| **3** | 1584 | 20.5 | 298 | 18.8 | 0.78 | 0.41 | 1.89 | 1.54-2.31 |  |
| **4** | 1353 | 17.5 | 348 | 25.7 | 0.60 | 0.62 | 2.82 | 2.31-3.44 |  |
| **5** | 1047 | 13.5 | 307 | 29.3 | 0.39 | 0.79 | 3.38 | 2.75-4.15 |  |
| **6** | 889 | 11.5 | 332 | 37.3 | 0.20 | 0.91 | 4.85 | 3.94-5.98 |  |
| **Total** | 7744 |  | 1658 |  |  |  |  |  |  |
|  |  |  |  |  |  |  |  |  |  |
|  | **Modified DIVERT 2** | | | | | | | |  |
|  |  |  |  |  |  |  |  |  |  |
|  | **Number of assessments** | | **Number of outcomes** | |  |  |  |  |  |
|  |  |  |  |  | **Sensitivity** | **Specificity** | **OR** | **95%CI** |  |
|  | **N** | **%** | **N** | **%** |  |  |  |  |  |
| **1** | 1591 | 20.5 | 174 | 10.9 |  |  | 1 |  |  |
| **2** | 1280 | 16.5 | 199 | 15.5 | 0.90 | 0.23 | 1.50 | 1.21-1.87 |  |
| **3** | 1710 | 22.1 | 339 | 19.8 | 0.78 | 0.41 | 2.01 | 1.65-2.45 |  |
| **4** | 1321 | 17.1 | 339 | 25.6 | 0.57 | 0.60 | 2.81 | 2.30-3.43 |  |
| **5** | 953 | 12.3 | 275 | 28.9 | 0.37 | 0.80 | 3.30 | 2.68-4.08 |  |
| **6** | 889 | 11.5 | 332 | 37.3 | 0.20 | 0.91 | 4.85 | 3.94-5.98 |  |
| **Total** | 7744 |  | 1658 |  |  |  |  |  |  |
