# Supplementary material for: The role of geriatric syndromes in predicting unplanned hospitalizations: a population-based study using Minimum Data Set for Home Care
Source: BMC Geriatr. 2023 Oct 26;23:696. doi: 10.1186/s12877-023-04408-w (PMC10605458; doi:10.1186/s12877-023-04408-w)
Supplement: Supplementary file 4 — Additional file 4. Sensitivity and specificity of the original (based on our earlier study using the same data 18) and modified DIVERT scales in the whole data and in different age groups. [file 12877_2023_4408_MOESM4_ESM.docx]

| **Additional file 4. Sensitivity and specificity of the original (based on our earlier study using the same data** ^18^**) and modified DIVERT scales in the whole data and in different age groups** | | | | | | | |  |
| --- | --- | --- | --- | --- | --- | --- | --- | --- |
|  |  |  |  |  |  |  |  |  |
|  |  |  |  |  |  |  |  |  |
| **DIVERT** | **Sensitivity** | | |  | **Specificity** | | |  |
| **LEVELS** | **Original** | **Modified 1** | **Modified 2** |  | **Original** | **Modified 1** | **Modified 2** |  |
| **1** | 1 | 1 | 1 |  |  |  |  |  |
| **2** | 0.90 | 0.9 | 0.90 |  | 0.23 | 0.23 | 0.23 |  |
| **3** | 0.68 | 0.78 | 0.78 |  | 0.50 | 0.41 | 0.41 |  |
| **4** | 0.50 | 0.60 | 0.57 |  | 0.69 | 0.62 | 0.60 |  |
| **5** | 0.30 | 0.39 | 0.37 |  | 0.83 | 0.79 | 0.80 |  |
| **6** | 0.15 | 0.20 | 0.20 |  | 0.93 | 0.91 | 0.91 |  |
|  |  |  |  |  |  |  |  |  |
| **<70 years** |  |  |  |  |  |  |  |  |
| **DIVERT** | **Sensitivity** | | |  | **Specificity** | | |  |
| **LEVELS** | **Original** | **Modified 1** | **Modified 2** |  | **Original** | **Modified 1** | **Modified 2** |  |
| **1** | 1 | 1 | 1 |  |  |  |  |  |
| **2** | 0.86 | 0.86 | 0.86 |  | 0.34 | 0.33 | 0.34 |  |
| **3** | 0.70 | 0.78 | 0.78 |  | 0.61 | 0.53 | 0.53 |  |
| **4** | 0.62 | 0.68 | 0.65 |  | 0.73 | 0.68 | 0.70 |  |
| **5** | 0.43 | 0.47 | 0.46 |  | 0.86 | 0.84 | 0.85 |  |
| **6** | 0.26 | 0.28 | 0.28 |  | 0.94 | 0.93 | 0.94 |  |
|  |  |  |  |  |  |  |  |  |
| **70-79 years** | |  |  |  |  |  |  |  |
| **DIVERT** | **Sensitivity** | | |  | **Specificity** | | |  |
| **LEVELS** | **Original** | **Modified 1** | **Modified 2** |  | **Original** | **Modified 1** | **Modified 2** |  |
| **1** | 1 | 1 | 1 |  |  |  |  |  |
| **2** | 0.91 | 0.91 | 0.91 |  | 0.27 | 0.27 | 0.27 |  |
| **3** | 0.72 | 0.79 | 0.79 |  | 0.53 | 0.45 | 0.45 |  |
| **4** | 0.54 | 0.63 | 0.61 |  | 0.68 | 0.63 | 0.60 |  |
| **5** | 0.30 | 0.35 | 0.33 |  | 0.82 | 0.79 | 0.80 |  |
| **6** | 0.18 | 0.22 | 0.22 |  | 0.93 | 0.90 | 0.91 |  |
|  |  |  |  |  |  |  |  |  |
| **80-89 years** | |  |  |  |  |  |  |  |
| **DIVERT** | **Sensitivity** | | |  | **Specificity** | | |  |
| **LEVELS** | **Original** | **Modified 1** | **Modified 2** |  | **Original** | **Modified 1** | **Modified 2** |  |
| **1** | 1 | 1 | 1 |  |  |  |  |  |
| **2** | 0.89 | 0.89 | 0.89 |  | 0.20 | 0.20 | 0.20 |  |
| **3** | 0.65 | 0.76 | 0.76 |  | 0.47 | 0.37 | 0.37 |  |
| **4** | 0.48 | 0.57 | 0.55 |  | 0.68 | 0.61 | 0.60 |  |
| **5** | 0.29 | 0.39 | 0.37 |  | 0.83 | 0.78 | 0.80 |  |
| **6** | 0.14 | 0.19 | 0.19 |  | 0.93 | 0.9 | 0.91 |  |
|  |  |  |  |  |  |  |  |  |
| **>90 years** |  |  |  |  |  |  |  |  |
| **DIVERT** | **Sensitivity** | | |  | **Specificity** | | |  |
| **LEVELS** | **Original** | **Modified 1** | **Modified 2** |  | **Original** | **Modified 1** | **Modified 2** |  |
| **1** | 1 | 1 | 1 |  |  |  |  |  |
| **2** | 0.90 | 0.90 | 0.90 |  | 0.16 | 0.15 | 0.16 |  |
| **3** | 0.69 | 0.81 | 0.81 |  | 0.44 | 0.33 | 0.33 |  |
| **4** | 0.48 | 0.61 | 0.58 |  | 0.67 | 0.57 | 0.60 |  |
| **5** | 0.30 | 0.40 | 0.37 |  | 0.80 | 0.74 | 0.76 |  |
| **6** | 0.13 | 0.20 | 0.20 |  | 0.93 | 0.88 | 0.88 |  |
